# Epilepsy-Related Brain Network Alterations in Patients With Temporal Lobe Glioma in the Left Hemisphere

**DOI:** 10.3389/fneur.2020.00684

**Published:** 2020-07-17

**Authors:** Shengyu Fang, Chunyao Zhou, Xing Fan, Tao Jiang, Yinyan Wang

**Affiliations:** ^1^Department of Neurosurgery, Beijing Neurosurgical Institute, Beijing, China; ^2^Department of Neurosurgery, Beijing Tiantan Hospital, Capital Medical University, Beijing, China

**Keywords:** functional network, glioma, graph theoretical analysis, resting-state fMRI, tumor-related epilepsy

## Abstract

**Background:** Seizures are a common symptom in patients with temporal lobe gliomas and may result in brain network alterations. However, brain network changes caused by glioma-related epilepsy (GRE) remain poorly understood.

**Objective:** In this study, we applied graph theory analysis to delineate topological networks with resting-state functional magnetic resonance images (rs-fMRI) and investigated characteristics of functional networks in patients with GRE.

**Methods:** Thirty patients with low-grade gliomas in the left temporal lobe were enrolled and classified into GRE (*n* = 15) and non-GRE groups. Twenty healthy participants matched for age, sex, and education level were enrolled. All participants had rs-fMRI data. Sensorimotor, visual, default mode, auditory, and right executive control networks were used to construct connection matrices. Topological properties of those sub-networks were investigated.

**Results:** Compared to that in the GRE group, four edges with higher functional connectivity were noted in the non-GRE group. Moreover, 21 edges with higher functional connectivity were identified in the non-GRE group compared to the healthy group. All significant alterations in functional edges belong to the visual network. Increased global efficiency and decreased shortest path lengths were noted in the non-GRE group compared to the GRE and healthy groups. Compared with that in the healthy group, nodal efficiency of three nodes was higher in the GRE and non-GRE groups and the degree centrality of six nodes was altered in the non-GRE group.

**Conclusion:** Temporal lobe gliomas in the left hemisphere and GRE altered visual networks in an opposing manner. These findings provide a novel insight into brain network alterations induced by GRE.

## Introduction

Seizures are a frequent symptom of brain tumors (1). Gliomas, particularly diffuse low-grade gliomas (DLGG, WHO grade 2), are highly epileptogenic (2). Most patients with DLGG experience glioma-related epilepsy (GRE) as a presenting symptom, especially for DLGGs growing in the temporal lobe (3). The prevailing view considers epilepsy to be a functional network disorder, and previous studies have reported correlations between alterations in functional networks and epileptic characteristics (4, 5). However, the etiology of GRE remains unclear.

Resting-state functional magnetic resonance imaging (rs-fMRI) enables quantitative transformation of functional connections, permitting delineation of brain networks. Graph theory analysis is a useful approach to quantitatively reveal the topological properties of brain networks (6, 7). Numerous studies have focused on the association between primary seizures and alterations in functional networks. Temporal lobe seizures induce functional connectivity (FC) and decreased network efficiency as seizures decrease intercortical synchronous fluctuations (6, 8, 9). However, alterations in functional networks induced by GRE are affected by both the glioma and GRE. Hence, previous conclusions regarding alterations in functional networks in primary seizures are insufficient, occluding appropriate preoperative prevention and intraoperative treatment. Moreover, the alterations in functional networks induced by temporal gliomas are unknown. Consequently, investigating the characteristics of functional network alterations induced by temporal GRE is critical to optimize preoperative prevention and intraoperative treatment.

To address this gap in knowledge, this study retrospectively enrolled 30 patients with left temporal DLGG (including 15 patients with glioma-related generalized seizures) and 20 healthy controls to investigate how temporal GRE altered functional networks. Our results indicated that temporal DLGG and GRE caused distinct alterations in brain networks.

## Methods

The local institutional review board approved this study. All participants provided written informed consent before data acquisition.

### Participants

Forty patients diagnosed with primary temporal lobe glioma and who had rs-fMRI data at Beijing Tiantan Hospital Glioma Treatment Center were recruited between January 2017 and July 2018. The patient inclusion criteria were as follows: (a) age ≥ 18 years, (b) histopathological diagnosis with primary DLGG according to the 2016 World Health Organization criteria; (c) more than 6 years of school education, and (d) no history of biopsy, radiotherapy, or chemotherapy. Exclusion criteria were as follows: (a) contraindications for MRI, or (b) head motion > 3 mm in translation or 3° in rotation. The healthy participant inclusion criteria were as follows: (a) age ≥ 18 years, (b) no history of brain disease, and (c) more than 6 years of school education. The exclusion criteria were as follows: (a) contraindications for MRI, or (b) head motion > 3 mm in translation or 3° in rotation.

### Clinical Characteristics Collection

We retrospectively collected patient characteristics from inpatient records, including age, sex, education level, Karnofsky performance status, histopathology, isocitrate dehydrogenase mutation status, extent of tumor resection, information regarding preoperative seizures, type of seizure onset, and history of taking anti-epileptic drugs. Follow-up information about postoperative epileptic control was obtained by telephone interviews at 6 months postoperatively.

### MRI Acquisition

A MAGNETOM Prisma 3-T MR scanner (Siemens, Erlangen, Germany) was used to acquire MR images. Anatomical images were collected by T1-magnetization prepared rapid acquisition gradient echo [repetition time [TR] = 2,300 ms; echo time [TE] = 2.3 ms; flip angle [FA] = 8°; field of view [FOV] = 220 × 220 mm; voxel size = 1.0 × 1.0 × 1.0 mm; slice number = 192]. A T2-FLAIR (fluid-attenuated inversion recovery) sequence was applied to acquire tumor images (TR = 3,200 ms; TE = 87 ms; FA = 150°; FOV = 220 × 220 mm; voxel size = 0.9 × 0.9 × 5 mm; slice number = 25). Additionally, the parameters for the rs-fMRI sequence were as follows: TR = 2,000 ms; TE = 30 ms; FA = 75°; FOV = 220 × 220 mm; voxel size = 3.0 × 3.0 × 5.0 mm; slice number = 30; acquisition duration: 8 min. All MRI data were acquired within 72 h before tumor resection.

### Functional MRI Preprocessing

The Graph Theoretical Network Analysis (GRETNA) toolbox (https://www.nitrc.org/projects/gretna) (10) was used for rs-fMRI processing. For each participant, pre-processing was conducted as follows: (a) transformation to a NIFTI file, (b) removal of the first images (time point number to remove = 5), (c) slice timing correction, (d) realignment, (e) spatial normalization [normalized to EPI template (11)], (f) smoothing (full width half maximum = 4 mm), (g) temporal detrending (linear detrending), (h) regressing out covariance (white matter signal: with WMMask_3 mm; CSF signal: with CSFMask_3 mm; head motion: Friston−24 parameters), (i) temporal filtering (0.01–0.08 Hz), and (j) scrubbing (using default parameters and the interpolation strategy: linear interpolation, FD threshold: 0.5, previous time point number: 1, subsequent time point number: 2).

### Regions of Tumor Invasion

Tumors were segmented into individual spaces based on hyper-intensive regions of FLAIR images. Regions of glioma invasion (shown in Figure S1) were manually drawn by two independent neuroradiologists. If the images drawn varied by more than 5%, a third neuroradiologist with over 20 years of clinical experience made the final decision regarding the region location. All tumor masks were then normalized into the Montreal Neurological Institute (MNI) standard space using the clinical toolbox package in SPM8 (http://www.fil.ion.ucl.ac.uk/spm/software/spm8).

### Regions of Interest

To calculate FC within cerebral functional networks, regions of interest (ROIs) were extracted from an open-access brain atlas, “brainnetome atlas” (http://www.brainnetome.org/) (12), which comprises 246 brain regions. In the current study, sub-templates were extracted, including sensorimotor, visual, default mode, auditory, and right executive control networks. Areas of tumor overlays were not included in the network analysis. Any potential effect of tumor invasion to registration would be highly decreased in this way. Details of each ROI are provided in Tables S1–S5.

### Network Construction

To construct the FC matrix, Pearson correlation coefficients were used to compare regional mean time-series for all possible pairs of nodes. Consequently, five different FC matrices were extracted from the five sub-templates of the sensorimotor, visual, default mode, auditory, and right executive control networks.

### Graph Theoretical Measures

Global and nodal topological properties, including the shortest path length, global efficiency, local efficiency, nodal efficiency, and degree centrality (DC), were calculated for all patients and healthy controls by using graph theory analysis. All matrices were transformed into absolute value and binary matrices before calculating topological properties.

Gamma, lambda, and sigma were indices of small worldness. Gamma (γ) = C_real_/C_random_ >> 1 (C represented cluster coefficient), lambda (λ) = L_real_/L_random_ ~ 1 (L represented shortest path length), sigma (σ) = γ/λ > 1 (13, 14). A high value of sigma indicates a high efficiency of information delivery.

### Statistical Analyses

Clinical characteristics were compared between the patient and healthy groups by using two-sample *t*-tests, Mann-Whitney U tests, chi-squared tests, one-way ANOVA tests, and Fisher's exact tests according to data type using GraphPad 7.0 (GraphPad Software; San Diego, CA).

Group differences in FC were, respectively, calculated based on each sub-template in GRETNA. To explore group differences in topological properties, we applied a series of sparsity thresholds (from 0.17 to 0.33, interval 0.01) consistent with a previously published study (4). For each participant, topological properties were calculated according to the corresponding FC matrix of each sub-template, which was generated according to sparsity. The areas under the curves of global and nodal topological characteristics were evaluated by means of one-way ANOVA (corrected with false-positive adjustment). *Post-hoc* pairwise comparisons for global and nodal characteristics were performed with two-sample *t*-tests. False discovery rate (FDR) was used to correct the differences in FC of each sub-template and nodal property.

### Data Availability Statement

Anonymized data will be made available on request.

## Results

### Demographic Characteristics

Of the 40 patients enrolled, 10 were excluded; hence, only the data of 30 patients were analyzed in the study. These 30 patients were classified into GRE (*n* = 15, 8 men) and non-GRE (*n* = 15, 7 men) groups based on the presence of GRE (Table 1). All patients were defined as right-handed using the Edinburgh Handedness Inventory test. The type of seizure onset was secondary generalized epilepsy. All patients accompanying glioma-related epilepsy (GRE) had taken levetiracetam 0.5 g twice a day to control GRE from the glioma diagnosed to surgery. There were four patients with GRE who took anti-epileptic drugs and experienced recurring epilepsy. Our postoperative follow-up showed that no patient with preoperative GRE experienced epilepsy at 6 months after tumor resection. All patients achieved Engel class I. In addition, 20 healthy participants matched for age, sex, and education level were recruited (9 men; all right-handed).

**Table 1:** Demographic and clinical characteristics of patient groups.

**Demographic and clinical characteristics**	**GRE (*n =* 15)**	**Non-GRE (*n =* 15)**	**Healthy (*n =* 20)**	***p*-value**
**Sex**				
Male	8	7	9	0.88
Female	7	8	11	
Age (y)^*^	38.2 ± 3.4	41.4 ± 3.1	38.0 ± 1.9	0.63
**Handness**				
Right	15	15	20	−
Left	0	0	0	
**KPS score (preoperative)**				
100	15	14	20	
90~100	0	1	0	> 0.99
80~90	0	0	0	
Education level (y)^*^	12.8 ± 1.2	13.2 ± 1.1	12.7 ± 0.8	0.13
**Histopathology**				
Astrocytoma	7	8	–	0.71
Oligodendroglioma	8	7	–	
**IDH status**				
Mutation	9	10		
Wild-type	6	5		0.71
Tumor volume (mL)^*^	44.83 ± 8.37	38.57 ± 10.16	–	0.64
Onset age (y)^*^	38.15 ± 1.9			–
**Frequency before diagnosis**				
Low (only once)	12			
Medium (2~3 times)	2			–
High (>3 times)	1			
**Preoperative anti-epileptic drugs**				–
Levetiracetam (0.5 g, twice a day)	15			
**Postoperative epileptic control**				–
Engel Class I	15			

* *Values are means ± standard deviations, unless indicated otherwise*.

*Using Mann-Whitney U test to compare differences in the Karnofsky performance status between the GRE and non- GRE groups*.

*Using Student's t-test to compare differences in tumor volume between the GRE and non-GRE groups*.

*Using one-way ANOVA test to compare differences in age and education level between the GRE and non-GRE groups*.

*Using chi-squared test to compare differences in sex, tumor location, and IDH status between the GRE and non-GRE groups*.

*IDH, isocitrate dehydrogenase; KPS, Karnofsky performance status*.

No significant differences were observed in age, sex, or years of education among the three groups (GRE, Non-GRE, control). No differences in Karnofsky performance status (*p* > 0.99, Mann-Whitney U test) or isocitrate dehydrogenase mutation status (*p* = 0.71, chi-squared test) were observed between the GRE and non-GRE groups. No significant differences in tumor volume were noted between the GRE and non-GRE groups (*p* = 0.64).

### Functional Connectivity Differences

FC was compared among GRE, non-GRE, and control groups in the matrices of sensorimotor, visual, default mode, auditory, and right executive control networks. Except for FC in the visual network, no significant differences in FC in the other five networks were noted after multiple correction.

**Figure 1:**
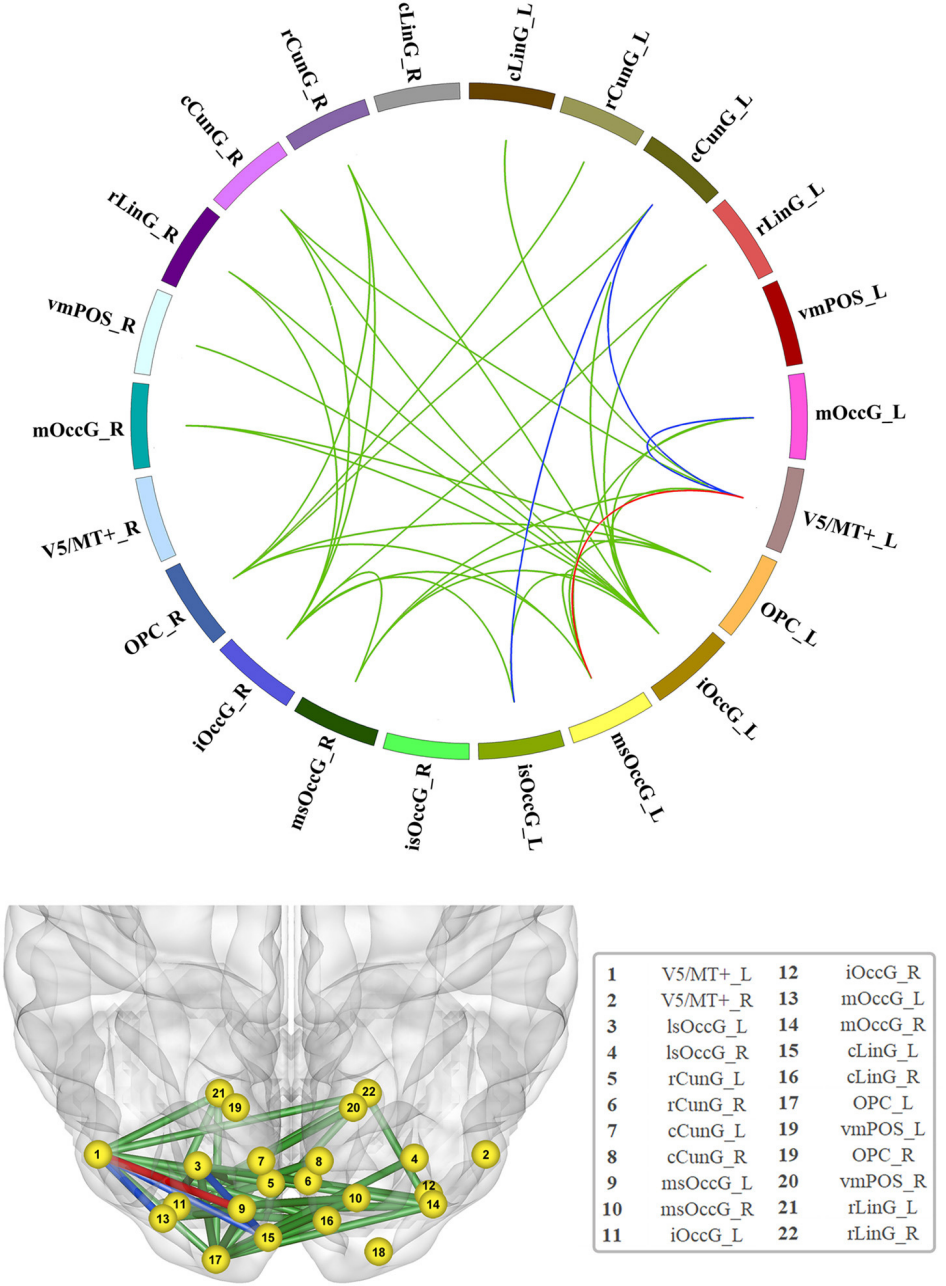
Significant increase in functional connectivity (FC). The red line represents the edge of FC that was increased in the non-GRE group compared to the GRE and healthy groups. The blue lines represent the edges of FC that were increased in the non-GRE group compared to the GRE group. The green lines represent the edges of FC that were increased in the non-GRE group compared to the healthy group.

In total, 231 functional edges belonged to the visual network. A significant difference in FC strength within the visual network among the three groups was observed after FDR correction (Figure 1 and detailed in Table S6).

Compared with those in the GRE group, four functional edges were identified with significantly higher FC in the non-GRE group after FDR correction (threshold of *p*-value = 6 × 10^−3^). Three of these edges originated from the V5/MT+_L node, which mediates sensory aspects of the visual motion area in the left hemisphere, and were connected to the medial superior occipital (msOccG_L), middle occipital (mOccG_L), and caudal cuneus gyri (cCunG_L) in the left hemisphere. The other edge connected the caudal cuneus gyrus (cCunG_L) to the lateral superior occipital gyrus (lsOccG_L) in the left hemisphere.

Compared with those in the control group, 21 functional edges were identified with significantly higher FC in the non-GRE group after FDR correction (threshold of *p*-value = 6 × 10^−3^, Table S7). Seven edges originated from the inferior occipital gyrus (iOccG_L) node in the left hemisphere and were connected to the bilateral caudal cuneus (cCunG_L/R), bilateral rostral lingual (rLinG_L/R), bilateral medial superior occipital (msOccG_L/R), and rostral cuneus gyri in the right hemisphere (rCunG_R). Six edges originated from the inferior occipital gyrus (iOccG_R) in the right hemisphere and were connected to the bilateral rostral lingual (rLinG_L/R), bilateral medial superior occipital (msOccG_L/R), ipsilateral caudal cuneus (cCunG_R), and contralateral rostral cuneus gyri (rCunG_R). Two edges originated from the occipital polar cortex node in the left hemisphere (OPC_L) and were connected to the bilateral medial superior occipital gyrus (msOccG_L/R). Four edges originated from the occipital polar cortex node in the right hemisphere (OPC_R) and were connected to the bilateral medial rostral cuneus (rCunG_L/R), contralateral caudal cuneus (cCunG_L), and contralateral medial superior occipital gyri (msOccG_L). Two edges originated from the node of V5/MT + in the left hemisphere (V5/MT + _L), and were connected to the ipsilateral medial superior occipital gyrus (msOccG_L/R) and caudal lingual gyrus (cLinG_L).

### Differences in Global Topological Properties

In the visual network, there were some differences in global efficiency (*p* = 0.025), shortest path length (*p* = 0.048), and vulnerability (*p* = 0.003) among patient and control groups by testing with one-way ANOVA.

**Figure 2:**
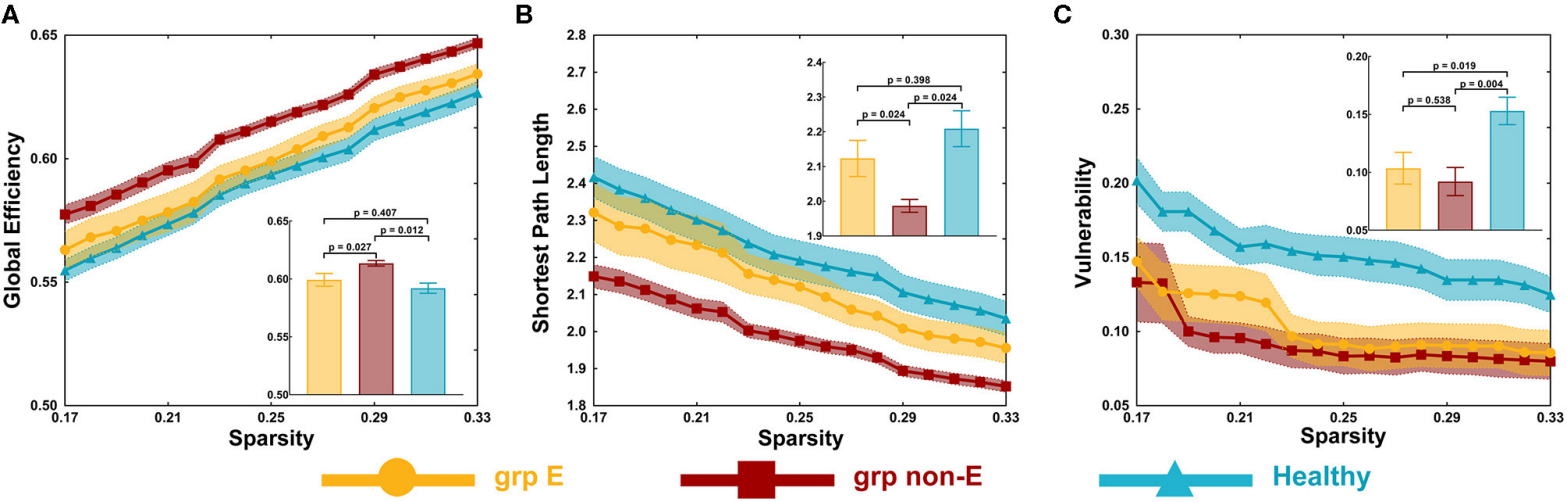
Global topological properties. **(A)** Global efficiency. **(B)** Shortest path length. **(C)** Vulnerability. *P*-value threshold = 0.05. Grp E = GRE group. Grp non-E = non-GRE group.

The non-GRE group exhibited significantly greater global efficiency (0.614 ± 0.002) than the GRE (0.599 ± 0.004, *p* = 0.013, *post-hoc* analysis, multiple correction with Tamhane's test) and control groups (0.592 ± 0.004, *p* < 0.001, *post-hoc* analysis, multiple correction with Tamhane's test, Figure 2A). No significant difference in global efficiency was detected between the GRE and control groups (*p* = 0.518, *post-hoc* analysis, multiple correction with Tamhane's test).

The non-GRE group exhibited a significantly shorter length of the shortest path (1.987 ± 0.019) than the GRE (2.123 ± 0.043, *p* = 0.039, *post-hoc* analysis, multiple correction with Tamhane's test, Figure 2B) and healthy groups (2.209 ± 0.052, *p* = 0.001, *post-hoc* analysis, multiple correction with Tamhane's test). No significant difference was observed between GRE and control groups (*p* = 0.385, *post-hoc* analysis, multiple correction with Tamhane's test).

The GRE group exhibited significantly worse network vulnerability (0.104 ± 0.014) than the healthy group (0.153 ± 0.012, *p* = 0.012, *post-hoc* analysis, multiple correction with Least Significance Difference, Figure 2C). The non-GRE group demonstrated significantly worse vulnerability (0.092 ± 0.012, *p* = 0.002, *post-hoc* analysis, multiple correction with Least Significance Difference) than the healthy group. No significant difference was noted between GRE and non-GRE groups (*p* = 0.606, *post-hoc* analysis, multiple correction with Least Significance Difference).

No significant alterations of small-worldness properties, including gamma, lambda, and sigma, were found among the three groups.

### Differences in Nodal Topological Properties

In the visual network, there were some differences in nodal efficiencies of the occipital polar cortex in the left hemisphere (OPC_L, *p* = 0.001), the occipital polar cortex in the right hemisphere (OPC_R, *p* = 0.003), and the inferior occipital gyrus in the left hemisphere (iOccG_L, *p* < 0.001) among patient and control groups by testing with one-way ANOVA. After *post-hoc* analysis, we found that compared with the healthy group, nodal efficiencies of the OPC_L, OPC_R, and iOccG_L of GRE and non-GRE groups significantly increased after multiple correction (Table S8). Hence, we respectively, compared differences in the nodal efficiency of these nodes between each of the patient and control groups.

**Figure 3:**
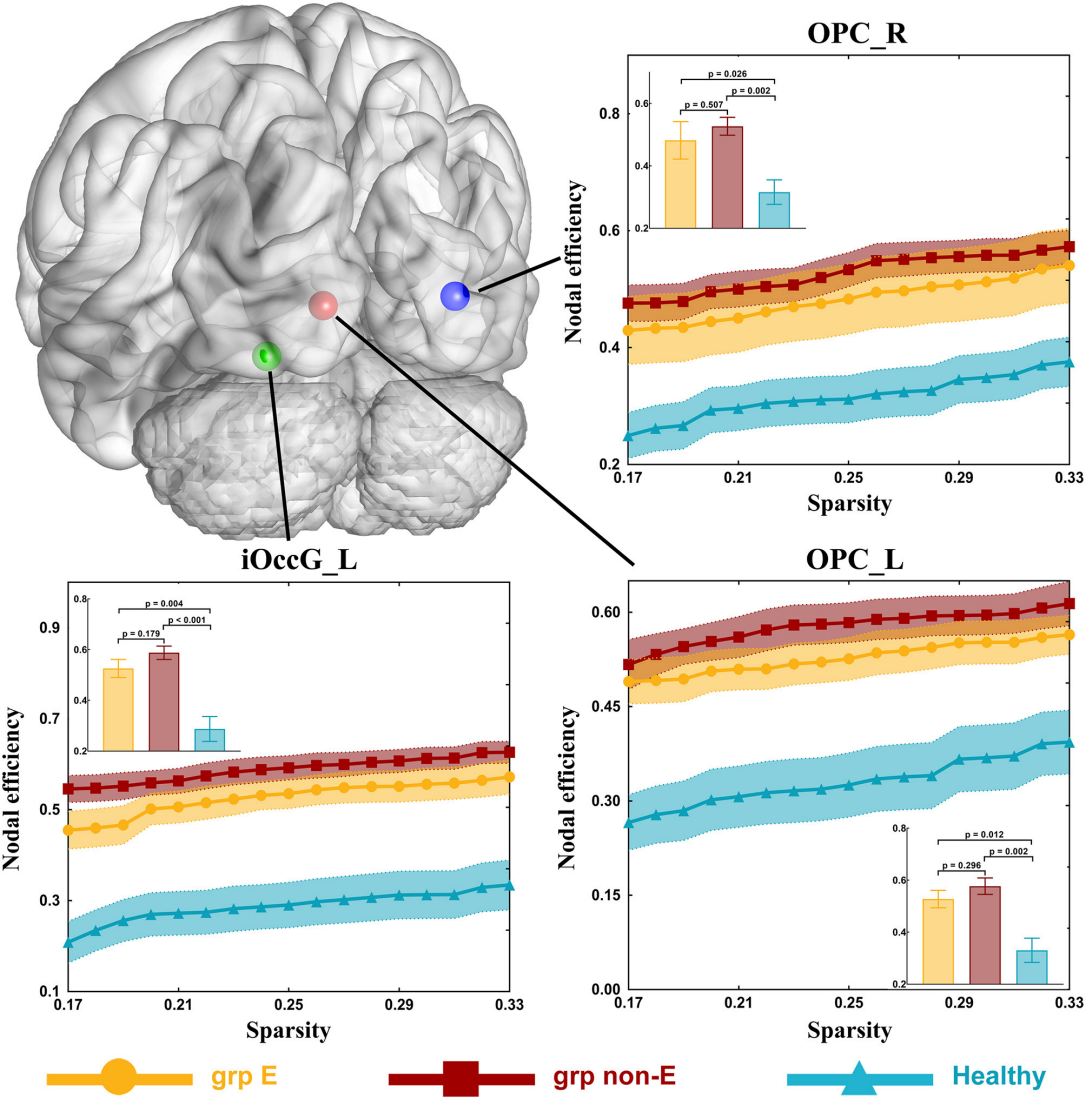
Significant alterations in nodal efficiency. Grp E = GRE group. *P*-value threshold after FDR correction is 0.002. Grp non-E = non-GRE group.

Nodal efficiencies of the OPC_L, OPC_R, and iOccG_L were significantly lower in the non-GRE group (OPC_L: 0.577 ± 0.032, OPC_R: 0.527 ± 0.029, and iOccG_L: 0.588 ± 0.026) than those in the control group (OPC_L: 0.330 ± 0.048, OPC_R: 0.316 ± 0.039, and iOccG_L: 0.287 ± 0.049) after FDR correction, respectively (OPC_L: *p* = 0.002, OPC_R: *p* = 0.002, and iOccG_L: *p* < 0.001, threshold of *p*-value = 0.002). Moreover, the nodal efficiencies of the OPC_L, OPC_R, and iOccG_L were lower in the GRE group than those in the control group, respectively, but these differences were not significant after FDR correction. Additionally, no differences were identified in nodal efficiency of the bilateral occipital polar cortex or inferior occipital gyrus between GRE and non-GRE groups (Figure 3 and Table S8).

**Figure 4:**
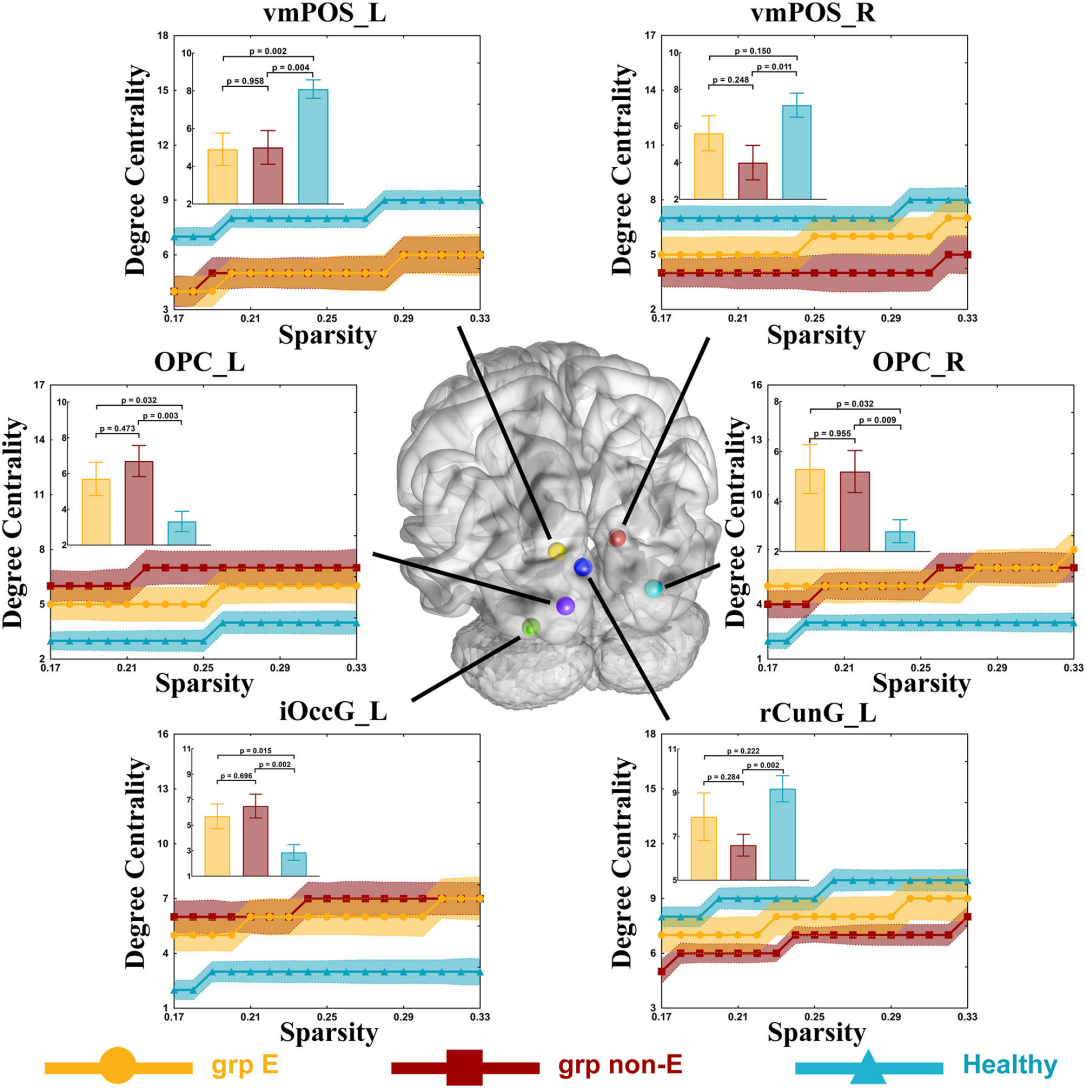
Significant alterations in degree centrality. Grp E = GRE group. *P*-value threshold after FDR correction is 0.011. Grp non-E = non-GRE group.

Compared to the control group (DC = 8.5), the DC of the ventromedial parieto-occipital sulcus node in the left hemisphere (vmPOS_L) was significantly lower in the GRE group (DC = 5, *p* = 0.002, Mann-Whitney U, FDR-corrected, *p*-value threshold = 0.011) and non-GRE group (DC = 5, *p* = 0.004, Mann-Whitney U, FDR-corrected, *p*-value threshold = 0.011). Compared with the control group (OPC_L: DC = 8.5, OPC_R: DC = 2.5), the DCs of occipital polar cortex nodes in the left (OPC_L), and right (OPC_R) hemispheres were significantly higher in the non-GRE group (OPC_L: DC = 6.5, *p* = 0.003, OPC_R: DC = 5, *p* = 0.009, Mann-Whitney U, FDR-corrected, *p*-value threshold = 0.011). Compared with the control group (DC = 2.5), the DC of the inferior occipital gyrus node in the left hemisphere (iOccG_L) was significantly higher in the non-GRE group (DC = 7, *p* = 0.002, Mann-Whitney U, FDR-corrected, *p*-value threshold = 0.011). Compared to the control group (vmPOS_R: DC = 8, rCunG_R: DC = 10), the DCs of the ventromedial parieto-occipital sulcus nodes in the right hemisphere (vmPOS_R) and the rostral cuneus gyrus in the left hemisphere (rCunG_L) were significantly lower in the non-GRE group (vmPOS_R: DC = 3, *p* = 0.011, and rCunG_L: DC = 7, *p* = 0.002, Mann-Whitney U, FDR-corrected, *p*-value threshold = 0.011, Figure 4 and Table S9). Relative to those of the GRE group, no differences were identified in nodal efficiency in the ventromedial parieto-occipital sulcus, rostral cuneus gyrus, and inferior occipital gyrus nodes in the right hemisphere or the bilateral occipital polar cortex between non-GRE and control groups.

## Discussion

This study explored alterations in functional networks induced by temporal GRE. Our findings indicated that both temporal glioma and GRE altered both FC and topological properties of the healthy visual network. No significant alterations caused by glioma and GRE were detected in other networks. We identified that temporal GRE facilitated alterations in the visual network in an opposite manner to that of glioma-induced alterations.

An extensive increase in visual network FC was induced by temporal glioma. This extensive increase in FC was not significant in patients with GRE compared to that in healthy controls. DLGG is a slow-growing primary brain tumor and is associated with marked functional plasticity (15). DLGG facilitates reorganization of functional networks adjacent to the tumor (16–18), resulting in increased FC (19). In our study, gliomas were located in the left temporal lobe, which is close to the visual network. Hence, visual network FC was increased in patients with glioma and without GRE. Conversely, primary epilepsy disrupts healthy networks by decreasing neuronal activity and synchronous fluctuations (5, 20–22), resulting in decreased FC of networks in patients with epilepsy. For this reason, visual network FC in patients with both temporal glioma and GRE was not significantly different from that of healthy controls. Based on our findings of FC alterations in the visual network, a hypothesis was proposed that GRE further alters the visual network based on alterations caused by temporal glioma.

Global efficiency represents the ability to integrate and communicate information (23). Increased global efficiency and decreased shortest path lengths in the visual network were noted in patients with temporal glioma. These changes were not significant when comparing patients with GRE to healthy controls. Reduced global efficiency has been reported in idiopathic epilepsy patients (24–28); however, our findings differed from these results. This discrepancy may result from differences in pathogenesis and network reorganization (16–18). As discussed above, a glioma induces reorganization of functional networks. Hence, the shortest path length was significantly decreased in patients with glioma and without GRE. Simultaneously, decreased shortest path lengths indicate alterations in structural connectivity and communication pathways. Temporal glioma-induced topological properties of the visual network were altered. Epilepsy can induce cortical sclerosis (29), gray matter atrophy (30), and cortical hypometabolism (31), resulting in decreased global efficiency and increased shortest path lengths in patients with epilepsy. Compared to that in healthy controls, global efficiency and shortest path lengths were not significantly altered in patients with temporal glioma and GRE. However, the absence of significant alterations does not necessarily indicate that GRE facilitated recovery of the visual network. We believe that this apparent lack of change is due to a combination of glioma and GRE. For instance, glioma increases global efficiency and GRE induces decreased global efficiency.

Vulnerability is an index to evaluate network stability (32). We observed that the vulnerability of patients in GRE and non-GRE groups were lower than that in the control group. These findings indicate that the visual network was more stable in patients than in controls. Simultaneously, our findings verified that both glioma itself and GRE induced alterations in the visual network, rather than GRE facilitating glioma-induced alterations in the visual network.

Alterations in topological properties of nodes highlighted in detail the form of network alterations. Compared with the control group, the nodal efficiencies of OPC_L, OPC_R, and iOccG_L were increased in the non-GRE group. Similarly, the DCs of these nodes had increased. These findings indicated that the OPC_L, OPC_R, and iOccG_L nodes were activated by the glioma itself and were inhibited by GRE. Moreover, the DC of the vmPOS_L node was significantly decreased in both GRE and non-GRE groups compared to the control group, suggesting that the glioma itself disrupted connections originating from vmPOS_L. Further, the DCs of vmPOS_R and rCunG_L nodes were reduced in the non-GRE group compared to the GRE and control groups. These DC alterations suggested that GRE altered the visual network by disrupting connections originating from these nodes.

Consequently, our findings verified that both temporal glioma and GRE altered the visual network, and the alterations caused by GRE were opposite to those caused by the glioma itself. Identifying the risk of preoperative seizures in patients with left temporal glioma requires analysis of nodal properties of nodes that were inhibited by GRE.

## Limitations

Although the number of patients was limited, the positive result identified in this situation should be reliable with a strict correction. In this study, the topological properties were calculated by using functional matrices with absolute values as previous studies used (14, 33, 34). Our findings showed that there was no difference in FC between the GRE and healthy groups. The phenomenon that aberrant FC in specific cognitive networks tended to normalize with levetiracetam administration was firstly found in patients with primary temporal epilepsy who took levetiracetam over 3 months (35). In our study, all patients in the GRE group took levetiracetam for a short period (<14 days). To our knowledge, it is still not clear that whether alterations of FC can be induced by levetiracetam in a short time. Future studies should focus on the time effect of antiepileptic drugs on brain networks.

## Conclusion

Temporal lobe glioma in the left hemisphere and GRE altered visual networks. Alterations in the visual network caused by GRE were opposite to those caused by the glioma itself. Our findings provide novel insight into GRE and contribute to improved understanding of functional network alterations in patients with glioma.

## Data Availability Statement

The datasets generated for this study are available on request to the corresponding author.

## Ethics Statement

The studies involving human participants were reviewed and approved by IRB of Beijing Tiantan Hospital. The patients/participants provided their written informed consent to participate in this study. Written informed consent was obtained from the individual(s) for the publication of any potentially identifiable images or data included in this article.

## Author Contributions

SF and CZ: study concept and design and data acquisition and analysis. SF, CZ, XF, and YW: statistics/verified analytical method. SF, CZ, and XF: writing the first draft. XF, YW, and TJ: supervision study. All authors read and approved final version. SF is the first contributed author of this manuscript. XF, YW, and TJ were all correspondent for this manuscript but not equally contributed authors.

## Conflict of Interest

The authors declare that the research was conducted in the absence of any commercial or financial relationships that could be construed as a potential conflict of interest.
