# Supplementary material for: Epilepsy-Related Brain Network Alterations in Patients With Temporal Lobe Glioma in the Left Hemisphere
Source: Front Neurol. 2020 Jul 17;11:684. doi: 10.3389/fneur.2020.00684 (PMC7380082; doi:10.3389/fneur.2020.00684)
Supplement: Supplementary file 1 [file Data_Sheet_1.DOCX]

# Supplemental Tables

**Supplementary Table 1. Montreal Neurological Institute (MNI) locations of 22 nodes in the sensorimotor network**

| **Regions of interesting** | **Modified Cyto-architectonic** | **Left hemisphere** | | | **Right hemisphere** | | |
| --- | --- | --- | --- | --- | --- | --- | --- |
|  |  | X | Y | Z | X | Y | Z |
| A6m_L(R) | Medial area BA 6 | 5 | 36 | 38 | 6 | 38 | 35 |
| A4hf_L(R) | Area BA 4 (head and face) | -49 | -8 | 39 | 55 | -2 | 33 |
| A4ul_L(R) | Area BA 4 (upper limb) | -26 | -25 | 63 | 34 | -19 | 59 |
| A4t_L(R) | Area BA 4 (trunk) | -13 | -20 | 73 | 15 | -22 | 71 |
| A4tl_L(R) | Area BA 4 (tongue and larynx) | -52 | 0 | 8 | 54 | 4 | 9 |
| A1_2_3ll_L(R) | Area BA 1/2/3 (lower limb) | -8 | -38 | 58 | 10 | -34 | 54 |
| A4ll_L(R) | Area BA 4 (lower limb) | -4 | -23 | 61 | 5 | -21 | 61 |
| A1_2_3ulhf_L(R) | Area BA 1/2/3 (upper limb and face) | -50 | -16 | 43 | 50 | -14 | 44 |
| A1_2_3tonIa_L(R) | Area BA 1/2/3 (tongue and larynx) | -56 | -14 | 16 | 56 | -10 | 15 |
| A2_L(R) | Area BA 2 | -46 | -30 | 50 | 48 | -24 | 48 |
| A1_2_3tru_L(R) | Area BA 1/2/3 (trunk) | -21 | -35 | 68 | 20 | -33 | 69 |

*****BA = Brodmann area.

**Supplementary Table 2. Montreal Neurological Institute (MNI) locations of 56 nodes in the default mode network.**

| **Regions of interesting** | **Modified Cyto-architectonic** | **Left hemisphere** | | | **Right hemisphere** | | |
| --- | --- | --- | --- | --- | --- | --- | --- |
|  |  | X | Y | Z | X | Y | Z |
| A8m_L(R) | medial area BA 8 | -5 | 15 | 54 | 7 | 16 | 54 |
| A8dl_L(R) | dorsolateral area BA 8 | -18 | 24 | 53 | 22 | 26 | 51 |
| A9l_L(R) | lateral area BA 9 | -11 | 49 | 40 | 13 | 48 | 40 |
| A6dl_L(R) | dorsolateral area BA 6 | -18 | -1 | 65 | 20 | 4 | 64 |
| A6m_L(R) | medial area BA 6 | -6 | -5 | 58 | 7 | -4 | 60 |
| A9m_L(R) | medial area BA 9 | -5 | 36 | 38 | 6 | 38 | 35 |
| A10m_L(R) | medial area BA 10 | -8 | 56 | 15 | 8 | 58 | 13 |
| A9_46d_L(R) | dorsal area BA 9/46 | -27 | 43 | 31 | 30 | 37 | 36 |
| A9_46v_L(R) | ventral area BA 9/46 | -41 | 41 | 16 | 42 | 44 | 14 |
| A8vl_L(R) | ventrolateral area BA 8 | -33 | 23 | 45 | 42 | 27 | 39 |
| A6vl_L(R) | ventrolateral area BA 6 | -32 | 4 | 55 | 34 | 8 | 54 |
| A20iv_L(R) | intermediate ventral area BA 20 | -45 | -26 | -27 | 46 | -14 | -33 |
| A37elv_L(R) | extreme lateroventral area BA 37 | -51 | -57 | -15 | 53 | -52 | -18 |
| A20r_L(R) | rostral area BA 20 | -43 | -2 | -41 | 40 | 0 | -43 |
| A20il_L(R) | intermediate lateral area 20 | -56 | -16 | -28 | 55 | -11 | -32 |
| A37vl_L(R) | ventrolateral area 37 | -55 | -60 | -6 | 54 | -57 | -8 |
| A20cl_L(R) | caudal lateral of area BA 20 | -59 | -42 | -16 | 61 | -40 | -17 |
| A20cv_L(R) | caudal ventral of area BA 20 | -55 | -31 | -27 | 54 | -31 | -26 |
| A35_36r_L(R) | rostral area BA 35/36 | -27 | -7 | -34 | 28 | -8 | -33 |
| A35_36c_L(R) | caudal area BA 35/36 | -25 | -25 | -26 | 26 | -23 | -27 |
| TL_R | posterior para-hippocampal gyrus | - | - | - | 30 | -30 | -18 |
| A28_34_L(R) | area BA 28/34, entorhinal cortex | -19 | -12 | -30 | 19 | -10 | -30 |
| TI_L(R) | temporal agranular insular cortex | - | - | - | 22 | 1 | -36 |
| TH_L(R) | medial posterior para-hippocampal gyrus | -17 | -39 | -10 | 19 | -36 | -11 |
| A5l_L(R) | lateral area BA 5 | -33 | -47 | 50 | 35 | -42 | 54 |
| A23d_L(R) | dorsal area BA 23 | -4 | -39 | 31 | 4 | -37 | 32 |
| A24rv_L(R) | rostroventral area BA 24 | -3 | 8 | 25 | 5 | 22 | 12 |
| A24cd_L(R) | caudal dorsal area BA 24 | -5 | 7 | 37 | 4 | 6 | 38 |
| A23c_L(R) | caudal area BA 23 | -7 | -23 | 41 | 6 | -20 | 40 |

*****BA = Brodmann area.

**Supplementary Table 3. Montreal Neurological Institute (MNI) locations of 4 nodes in the auditory network.**

| **Regions of interesting** | **Modified Cyto-architectonic** | **Left hemisphere** | | | **Right hemisphere** | | |
| --- | --- | --- | --- | --- | --- | --- | --- |
|  |  | X | Y | Z | X | Y | Z |
| A41/42_R | BA 41/42 | - | - | - | 54 | -24 | 11 |
| TE_R | TE1.0 and TE1.2 | - | - | - | 51 | -4 | -1 |
| A40rv_L(R) | rostroventral area 40 (PFop) | -53 | -31 | 23 | 55 | -26 | 26 |

*****BA = Brodmann area.

**Supplementary Table 4. Montreal Neurological Institute (MNI) locations of 22 nodes in the visual network.**

| **Regions of interesting** | **Modified Cyto-architectonic** | **Left hemisphere** | | | **Right hemisphere** | | |
| --- | --- | --- | --- | --- | --- | --- | --- |
|  |  | X | Y | Z | X | Y | Z |
| cLinG_L(R) | caudal lingual gyrus | -11 | -82 | -11 | 10 | -85 | -9 |
| rCunG_L(R) | rostral cuneus gyrus | -5 | -81 | 10 | 7 | -76 | 11 |
| cCunG_L(R) | caudal cuneus gyrus | -6 | 94 | 1 | 8 | -90 | 12 |
| rLinG_L(R) | rostral lingual gyrus | -17 | -60 | -6 | 18 | -60 | -7 |
| vmPOS_L(R) | ventromedial parietal occipital sulcus | -13 | -68 | 12 | 15 | -63 | 12 |
| mOccG_L(R) | middle occipital gyrus | -31 | -89 | 11 | 34 | -86 | 11 |
| V5/MT+_L(R) | area V5/MT+ | -46 | -74 | 3 | 48 | -70 | -1 |
| OPC_L(R) | occipital polar cortex | -18 | -99 | 2 | 22 | -97 | 4 |
| iOccG_L(R) | inferior occipital gyrus | -30 | -88 | -12 | 32 | -85 | -12 |
| msOccG_L(R) | medial superior occipital gyrus | -11 | -88 | 31 | 16 | -85 | 34 |
| lsOccG_L(R) | lateral superior occipital gyrus | -22 | -77 | 36 | 29 | -75 | 36 |

*****BA = Brodmann area.

**Supplementary Table 5. Montreal Neurological Institute (MNI) locations of 12 nodes in the right executive control network.**

| **Regions of interesting** | **Modified Cyto-architectonic** | **Right hemisphere** | | |
| --- | --- | --- | --- | --- |
|  |  | X | Y | Z |
| A8dl_R | dorsolateral area BA 8 | 22 | 26 | 51 |
| A9m_R | medial area BA 9 | 6 | 38 | 35 |
| A9/46d_R | dorsal area BA 9/46 | 30 | 37 | 36 |
| IFJ_R | inferior frontal junction | 42 | 11 | 39 |
| A46_R | area BA 46 | 28 | 55 | 17 |
| A9/46v_R | ventral area BA 9/46 | 42 | 44 | 14 |
| A8vl_R | ventrolateral area BA 8 | 42 | 27 | 39 |
| A10l_R | ventrolateral area BA 6 | 34 | 8 | 54 |
| A7ip_ R | intraparietal area BA 7 (hIP3) | 31 | -54 | 53 |
| A39rd _R | rostral dorsal area BA 39 (Hip3) | 39 | -65 | 44 |
| A40c_ R | caudal area BA 40 (PFm) | 57 | -44 | 38 |
| A39rv_R | rostroventral area BA 39 (PGa) | 53 | -54 | 25 |

*****BA = Brodmann area

**Supplementary Table 6. Functional connections with significant group effects between the GRE and non-GRE groups**

| **Connections** | **Functional connectivity (mean ± SEM)** | | ***p* value**  **(threshold of *p* value = 0.006)** |
| --- | --- | --- | --- |
|  | GRE | non-GRE |  |
| mOccG_L and V5/MT+_L | 0.551 ± 0.098 | 1.015 ± 0.059 | 0.0003 |
| cCunG_L and V5/MT+_L | 0.225 ± 0.098 | 0.658 ± 0.070 | 0.0022 |
| msOccG_L and V5/MT+_L | 0.519 ± 0.110 | 1.034 ± 0.079 | 0.0011 |
| cCunG_L and lsOccG_L | 0.308 ± 0.069 | 0.699 ± 0.079 | 0.0009 |

***** mOccG_L = middle occipital gyrus in the left hemisphere; cCunG_L = caudal cuneus gyrus in the left hemisphere; V5/MT+_L = area V5/MT+ in the left hemisphere; msOccG_L = medial superior occipital gyrus in the left hemisphere; lsOccG_L = lateral superior occipital gyrus in the left hemisphere. GRE = group of patients with glioma-related epilepsy; non-GRE = group of patients without glioma-related epilepsy.

**Supplementary Table 7. Functional connections with significant group effects between the non-GRE and healthy groups**

| **Connections** | **Functional connectivity (mean ± SEM)** | | ***p* value**  **(threshold of *p* value = 0.006)** |
| --- | --- | --- | --- |
|  | non-GRE | Healthy |  |
| cLinG_L and V5/MT+_L | 0.868 ± 0.06 | 0.530 ± 0.04 | 0.0003 |
| rCunG_L and OPC_R | 0.850 ± 0.08 | 0.443 ± 0.05 | 0.0003 |
| rCunG_R and OPC_R | 0.859 ± 0.09 | 0.414 ± 0.06 | 0.0004 |
| rCunG_R and iOccG_L | 0.797 ± 0.11 | 0.426 ± 0.05 | 0.0006 |
| rCunG_R and iOccG_R | 0.789 ± 0.07 | 0.473 ± 0.05 | 0.0014 |
| cCunG_L and OPC_R | 1.027 ± 0.12 | 0.595 ± 0.06 | 0.0020 |
| cCunG_L and iOccG_L | 0.865 ± 0.07 | 0.505 ± 0.05 | 0.0010 |
| cCunG_R and iOccG_L | 0.858 ± 0.07 | 0.475 ± 0.05 | 0.0004 |
| cCunG_R and iOccG_R | 0.808 ± 0.07 | 0.508 ± 0.04 | 0.0014 |
| rLinG_L and iOccG_L | 0.822 ± 0.09 | 0.385 ± 0.05 | 0.0001 |
| rLinG_L and iOccG_R | 0.741 ± 0.09 | 0.389 ± 0.04 | 0.0006 |
| rLinG_R and iOccG_L | 0.743 ± 0.09 | 0.340 ± 0.05 | 0.0005 |
| rLinG_R and iOccG_R | 0.729 ± 0.10 | 0.361 ± 0.05 | 0.0005 |
| V5/MT+_L and msOccG_L | 1.034 ± 0.08 | 0.608 ± 0.04 | <0.0001 |
| OPC_L and msOccG_L | 0.560 ± 0.09 | 0.370 ± 0.05 | 0.0004 |
| OPC_L and msOccG_R | 0.660 ± 0.08 | 0.324 ± 0.04 | 0.0005 |
| OPC_R and msOccG_L | 0.718 ± 0.09 | 0.352 ± 0.05 | 0.0007 |
| iOccG_L and msOccG_L | 0.785 ± 0.11 | 0.415 ± 0.05 | 0.0019 |
| iOccG_L and msOccG_R | 0.786 ± 0.11 | 0.361 ± 0.05 | 0.0004 |
| iOccG_R and msOccG_L | 0.836 ± 0.10 | 0.447 ± 0.04 | 0.0003 |
| iOccG_R and msOccG_R | 0.832 ± 0.11 | 0.411 ± 0.04 | 0.0001 |

***** cLinG_L = caudal lingual gyrus in the left hemisphere; rCunG_L(R) = rostral cuneus gyrus in the left (right) hemisphere; cCunG_L(R) = caudal cuneus gyrus in the left (right) hemisphere; rLinG_L(R) = rostral lingual gyrus in the left (right) hemisphere; V5/MT+_L = area V5/MT+ in the left hemisphere; iOccG_L(R) = inferior occipital gyrus in the left (right) hemisphere; msOccG_L(R) = medial superior occipital gyrus in the left (right) hemisphere; non-GRE = group of patients without glioma-related epilepsy.

**Supplementary Table 8. Difference of nodal efficiency among the** **epileptic,** **non-epileptic, and control groups.**

| **Nodes** | **Nodal efficiency (mean ± SEM)** | | | **One-way ANOVA**  **(*p* value)** | **Post-hoc analysis**  **(*p* value)** | | | | **Two sample t test**  **(*p* value, threshold of *p* = 0.002)** | | |
| --- | --- | --- | --- | --- | --- | --- | --- | --- | --- | --- | --- |
|  | **GRE**  **(n = 15)** | **non-GRE**  **(n = 15)** | **Healthy**  **(n = 20)** |  | **GRE vs non-GRE** | **GRE vs Healthy** | | **non-GRE vs Healthy** | **GRE vs non-GRE** | **GRE vs Healthy** | **non-GRE vs Healthy** |
| OPC_L | 0.527 ± 0.033 | 0.577 ± 0.032 | 0.330 ± 0.047 | 0.001 | 0.530 | | 0.005 | 0.001 | 0.296 | 0.012 | 0.002 |
| OPC_R | 0.482 ± 0.060 | 0.527 ± 0.029 | 0.316 ± 0.039 | 0.003 | 0.554 | | 0.014 | 0.002 | 0.507 | 0.026 | 0.002 |
| iOccG_L | 0.526 ± 0.036 | 0.588 ± 0.026 | 0.287 ± 0.049 | < 0.001 | 0.450 | | 0.001 | < 0.001 | 0.179 | 0.004 | < 0.001 |

***** GRE = group of patients with glioma-related epilepsy; non-GRE = group of patients without glioma-related epilepsy; OPC_L = occipital polar cortex in the left hemisphere; OPC_R= occipital polar cortex in the right hemisphere; iOccG_L = inferior occipital gyrus in the left hemisphere. Post hoc analysis was multiply corrected with Least Significance Difference. Two sample t test was multiply corrected by False Discover Rate.

**Supplementary Table 9. Difference of degree centrality among the GRE, non-GRE, and healthy groups.**

| **Nodes** | **Degree centrality (mean)** | | | ***P* value**  **(threshold of *p* = 0.011)** | | |
| --- | --- | --- | --- | --- | --- | --- |
|  | **GRE**  **(n = 15)** | **non-GRE**  **(n = 15)** | **Healthy**  **(n = 20)** | **GRE vs non-GRE** | **GRE vs Healthy** | **non-GRE vs Healthy** |
| OPC_L | 5 | 6.5 | 3 | 0.473 | 0.032 | 0.003 |
| OPC_R | 6.5 | 4.5 | 2.5 | 0.955 | 0.032 | 0.009 |
| iOccG_L | 6 | 7 | 2.5 | 0.696 | 0.015 | 0.002 |
| vmPOS_L | 5 | 5 | 8.5 | 0.958 | 0.002 | 0.004 |
| vmPOS_R | 6 | 3 | 8 | 0.248 | 0.150 | 0.011 |
| rCunG_L | 8.5 | 7 | 10 | 0.284 | 0.222 | 0.002 |

* OPC_L = occipital polar cortex in the left hemisphere; OPC_R= occipital polar cortex in the right hemisphere; iOccG_L = inferior occipital gyrus in the left hemisphere; vmPOS_L = ventromedial parietal-occipital sulcus in the left hemisphere; vmPOS_R = ventromedial parietal-occipital sulcus in the right hemisphere; rCunG_L = rostral cuneus gyrus in the left hemisphere. GRE = group of patients with glioma-related epilepsy; non-GRE = group of patients without glioma-related epilepsy.

# Supplemental Figure

**
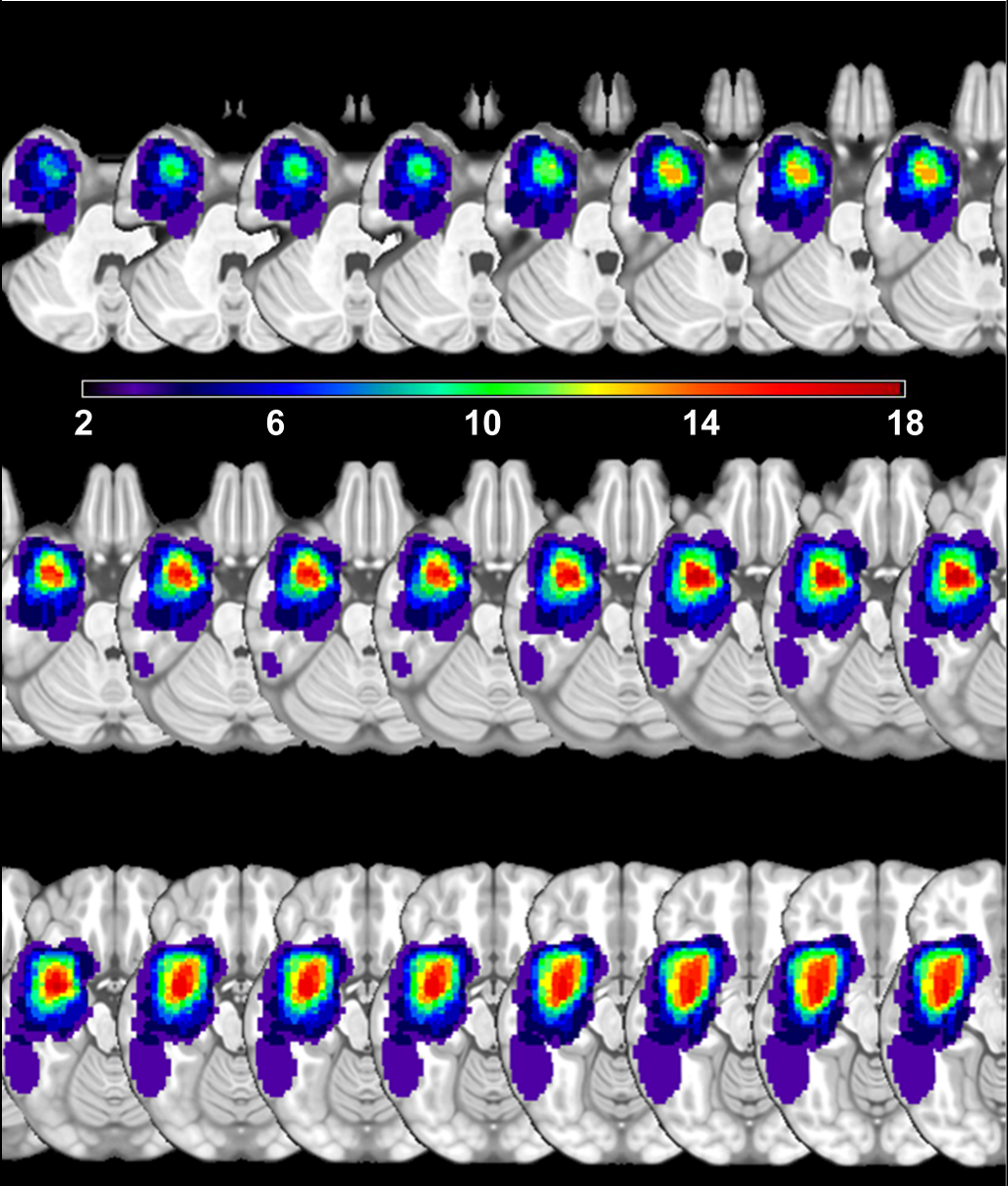
**

**Figure. Sup-1.** The overlapping results of temporal lobe gliomas. The value of color bar represents the number of patients with tumor located in a same region.
